# Pain Complaints and Intubation Risk in COVID-19: A Retrospective Cohort Study

**DOI:** 10.7759/cureus.33851

**Published:** 2023-01-16

**Authors:** Connor Martin, Oluseyi Obadeyi, Elizabeth Yeo, Duc Tran, Eugene Pak

**Affiliations:** 1 Physical Medicine and Rehabilitation, Loma Linda University Medical Center, Loma Linda, USA; 2 Emergency, Loma Linda University Medical Center, Loma Linda, USA; 3 Pain Medicine, Loma Linda University Medical Center, Loma Linda, USA

**Keywords:** sars-cov-2 (severe acute respiratory syndrome coronavirus 2), covid-gram, intubation, abdominal pain, covid-19

## Abstract

Background

Since coronavirus disease 2019 (COVID-19) emerged, increasing cases have been identified worldwide. COVID-19 continues to lead to significant morbidity and mortality, despite developing a vaccination for the disease. While much has been studied regarding the initial presentation and treatment of patients with COVID-19, to our knowledge, no study has uncovered that COVID-19-positive patients with abdominal pain are at a higher risk of requiring intubation.

Methodology

In this retrospective cohort study, we identified 104 patients who presented to the emergency room of a single tertiary care center with laboratory-confirmed COVID-19 between February 1, 2020, and April 27, 2020, and collected data on reported pain complaints.

Results

In this retrospective cohort study, the most common pain complaints were chest pain (25.5%), myalgia (23.4%), and abdominal pain (17.0%). Less common pain complaints included headaches (14.9%) and neck/back pain (6.3%). Of these pain complaints, only patients who reported having abdominal pain were more likely to be intubated (37.5% of patients with abdominal pain were intubated compared to 8.3% of patients without abdominal pain, with a p-value of 0.001).

Conclusions

Abdominal pain in a patient with COVID-19 infection significantly increases their chances of requiring intubation based on the results of this study.

## Introduction

In December 2019, numerous cases of pneumonia with unknown etiology emerged in Wuhan, China. These cases arose from a novel coronavirus identified as severe acute respiratory syndrome coronavirus 2 (SARS-CoV-2). The disease, coronavirus disease 2019 (COVID-19), was soon declared a global pandemic by the World Health Organization (WHO) on March 11, 2020 [1]. Since the first case was reported in China in 2019, significant progress has been made regarding the slowing of transmission, detection of disease, treatment of those infected with COVID-19, and even prevention of COVID-19 with the emergence of a vaccination. Despite the development of various types of vaccination, COVID-19 cases remain prevalent and with occasional surges of deadlier or more transmissible variants (SARS-CoV-2 variants, spike mutations, and immune escape). As of September 2022, COVID-19 cases have surpassed 97 million in the United States and 620 million worldwide, leading to significant morbidity and mortality [2].

While much focus has been placed on respiratory symptoms, underlying medical comorbidities, and age when predicting disease severity, few studies have addressed the implications of pain reported by patients with COVID-19. In our proposed research, we identified COVID-19-positive patients and investigated if their presenting pain symptoms were associated with worse outcomes.

## Materials and methods

Study design and participation

In this retrospective cohort study, we identified 104 patients who presented to the emergency department of a single tertiary care center in Southern California. These patients presented with a nasopharyngeal swab polymerase chain reaction (PCR) test positive for COVID-19 between February 1, 2020, and April 27, 2020. Clinical retrospective data were obtained from our electronic medical records, including demographic features, clinical features, and treatment interventions. A team of trained triage nurses recorded patients’ presenting symptoms. A trained team of physicians reviewed the data to determine patient eligibility in the study. Of the 104 patients identified, 94 were included in the study. Inclusion criteria included every patient with a laboratory-confirmed positive COVID-19 test result. Exclusion criteria included patients below 18 years old, nonverbal at baseline, or unconscious at initial presentation.

Statistical analysis

Categorical variables were expressed as a frequency with a percentage. Continuous variables were described as a mean with standard deviation. Proportions for categorical variables were compared using the chi-square test. Logistic regression was used for continuous data with one independent variable, and multiple logistic regression was used for continuous data with two or more independent variables. Statistical analysis was performed using Stata (StataCorp LLC, College Station, TX, USA). A p-value of <0.05 was considered statistically significant.

## Results

Of the 94 patients included in the study, the mean age was 51.6 ± 19.7 years, and 52 (55.3%) were females. The most prevalent race was White (Hispanic) (44%), followed by White (non-Hispanic) (27%), Black (17%), and Asian (9%). Of these patients, 61.7% required admission to the hospital, and 12.7% required intubation (Table 1).

**Table 1:** Demographic and baseline characteristics with outcome measure. *: One patient intubated had both myalgia and abdominal pain.

Intubated	All patients (N = 94)	Not intubated (N = 82)	Intubated (N = 12)	P-value
Age, years, mean (SD)	51.6 (19.7)	50.0 (19.1)	62.7 (21.2)	0.044
Sex (M/F)				
Male	42 (44.7%)	35 (42.7%)	7 (58.3%)	
Female	52 (55.3%)	47 (57.3%)	5 (41.7%)	
Race				
White (non-Hispanic)	25 (26.6%)	21 (25.6%)	4 (33.3%)	0.927
White (Hispanic)	42 (44.7%)	35 (42.7%)	6 (50.0%)	
Black	16 (17.0%)	15 (18.3%)	1 (8.3%)	
Asian	8 (8.5%)	7 (8.5%)	1 (8.3%)	
Native American/Pacific Islander	2 (2.1%)	2 (2.4%)	0 (0.0%)	
Unknown	1 (1.1%)	1 (1.1%)	0 (0.0%)	
Admission status				
Admitted to hospital	58 (61.7%)	46 (56.1%)	12 (100%)	0.003
Discharge from the emergency department	36 (38.3%)	36 (43.9%)	0 (0.0%)	
Presence of pain				
Single pain complaint	34% (36.2%)	28 (34.1%)	6 (50.0%)	0.385
Multiple pain complaints	22 (23.4%)	21 (25.6%)	1 (8.3%)	
No pain	38 (40.4%)	33 (40.2%)	5 (41.7%)	
Specific pain complaint				
Chest pain	24 (25.5%)	23 (28.0%)	1 (8.3%)	0.285
Myalgia	22 (23.4%)	21 (25.6%)	1 (8.3%)*	0.283
Headache	14 (14.9%)	14 (17.1%)	0 (0.0%)	0.202
Abdominal pain	16 (17.0%)	10 (12.2%)	6 (50.0%)	0.001
Neck/Back pain	6 (6.4%)	6 (7.3%)	0 (0.0%)	0.333
Discharge status				
Alive	87 (92.3%)	82 (100.0%)	5 (41.7%)	0.001
Deceased	7 (7.4%)	0 (0.0%)	7 (58.3%)	

We collected data on five different pain complaints. The most common pain complaints were chest pain (25.5%), myalgia (23.4%), and abdominal pain (17.0%). Less common pain complaints included headaches (14.9%) and neck/back pain (6.3%). Older patients were statistically more likely to be intubated than younger patients (p = 0.044). Patients who reported having abdominal pain had a worse outcome (37.5% of patients with abdominal pain were intubated compared to 8.3% of patients without abdominal pain, with a p-value of 0.001).

Table 2 shows pain complaints by age group. Chest and abdominal pain were more likely in the younger patient populations compared to the older (Table 2). Overall, there was an inverse relationship between age and pain complaints, with older patients reporting less pain (p = 0.033). Patients between the ages of 19-39 years had the highest frequency of pain complaints.

**Table 2:** Pain complaint by age group.

Age (years)	0–18 (N = 6)	19–39 (N = 20)	40–65 (N = 41)	66+ (N = 27)	P-value
Presence of pain					
Pain present	4 (66.7%)	15 (75.0%)	26 (63.4%)	11 (40.7%)	0.033
No pain present	2 (33.3%)	5 (25.0%)	15 (36.6%)	16 (59.3%)	
Type of pain					
Chest pain	1 (16.7%)	11 (55.0%)	11 (26.8%)	1 (3.7%)	0.005
Myalgia	0 (0.0%)	8 (40.0%)	11 (26.8%)	3 (11.1%)	0.285
Headache	2 (33.3%)	6 (30%)	4 (9.8%)	2 (7.4%)	0.020
Abdominal pain	2 (33.3%)	2 (10.0%)	7 (17.1%)	5 (18.5%)	0.962
Neck/Back pain	0 (0.0%)	0 (0.0%)	4 (9.8%)	2 (7.4%)	0.268

Table 3 shows a sub-analysis of pain complaints by race. The only statistically significant finding of pain complaints across races was that Native Americans/Pacific Islanders reported more headaches. Chest pain and myalgia were the two most common pain complaints across races. Asians and Hispanic Whites were more likely to report abdominal pain as a presenting pain complaint (Table 3).

**Table 3:** Pain complaint by race.

Race	White (non-Hispanic) (N = 25)	White (Hispanic) (N = 42)	Black (N = 16)	Asian (N = 8)	Native American/Pacific Islander (N = 2)	Unknown (N = 1)	P-value
Presence of pain							
Pain present	16 (64.0%)	24 (57.1%)	8 (50.0%)	5 (62.5%)	2 (100.0%)	1 (100.0%)	0.844
No pain present	9 (36.0%)	18 (42.9%)	8 (50.0%)	3 (37.5%)	0 (0.0%)	0 (0.0%)	
Type of pain							
Chest pain	7 (28.0%)	11 (26.2%)	4 (25.0%)	0 (0.0%)	1 (50.0%)	1 (100%)	0.242
Myalgia	7 (28.0%)	7 (16.7%)	5 (31.3%)	2 (25.0%)	1 (50%)	0 (0.0%)	0.590
Headache	2 (8.0%)	6 (14.3%)	3 (18.8%)	0 (0.0%)	2 (100%)	1 (100%)	0.012
Abdominal pain	4 (16.0%)	9 (21.4%)	1 (6.3%)	2 (25.0%)	0 (0.0%)	0 (0.0%)	0.718
Neck/Back pain	2 (8.0%)	2 (4.8%)	1 (6.3%)	1 (12.5%)	0 (0.0%)	0 (0.0%)	0.749

Table 4 shows the number of abdominal pathologies found in patients with abdominal pain and the type of image modality. Seven of the 16 patients who presented with abdominal pain received imaging, while nine did not. Three of the seven patients with abdominal pain who received imaging were intubated in the emergency department and eventually passed away during hospitalization.

**Table 4:** Abdominal pathologies found in patients with abdominal pain and the type of image modality utilized.

Abdominal pathology	Image modality
Biliary sludge without evidence of cholelithiasis or cholecystitis	Abdominal ultrasound
Nonobstructive bowel gas pattern	kidney, ureter, and bladder radiograph
Ileal neoplasm complicated by perforation and multifocal abscess formation versus less likely adnexal origin	CT scan of the abdomen and pelvis with intravenous contrast
No evidence of acute intra-abdominal/intrapelvic abnormality	CT scan of the abdomen and pelvis with intravenous contrast
Mild dilatation of the common bile duct without visualization of choledocholithiasis	Abdominal ultrasound
Deep vein thrombosis extends from the lower inferior vena cava to the visualized bilateral lower extremity veins	CT scan of the abdomen and Pelvis with intravenous contrast
Nonspecific gaseous distention of large bowel with prominent stool volume	CT scan of the abdomen and pelvis with intravenous contrast

## Discussion

This report further adds to the limited knowledge regarding COVID-19 and delves deeper into the significance of pain reported by patients with COVID-19. The study included patients seen during the initial period of the pandemic to focus on the first COVID-19 strain before other mutated variants became prevalent. Based on this data, abdominal pain in suspected or confirmed COVID-19 patients has significant prognostic implications. Abdominal pain significantly increases a patient’s chances of requiring intubation based on the above results (Table 2). This is one of the first studies to examine intubation specifically. A pooled analysis (n = 731) including three separate studies found an almost four-fold increase in the odds of severe COVID-19 with abdominal pain. It has been hypothesized that abdominal pain may be related to viral replication in the gut, with increased severity of illness observed in association with a high viral load and subsequent viremia [3,4]. In addition, pathologic changes have been reported at autopsy, further linking abdominal pain with severe disease. These include segmental dilation and stenosis of the small intestine and necrosis of the gastrointestinal mucosa on histology [5]. Studies have identified COVID-19 in the stool of infected patients, and its viral receptor, angiotensin-converting enzyme 2 (ACE2), is highly expressed in gastrointestinal epithelial cells [6,7]. Other studies have reported additional gastrointestinal symptoms, including diarrhea, vomiting, and anorexia [8]. Several different risk scores to help predict critical illness have been used in clinical practice for those diagnosed with COVID-19 [9]. The COVID-GRAM was validated with a cohort of 710 patients to predict critical illness but failed to include abdominal pain as a prognostic factor as abdominal pain was not found to be an independent statistically significant predictor of acute disease among hospitalized COVID-19-infected patients [10].

Pain complaints were reported less in the older populations than in younger ones (Table 2). This finding is consistent with other research done in aging populations concluding that nociception, or the perception of pain in response to painful stimuli, decreases with advancing age [11]. A meta-analysis investigating aging and changes in pain perception found that reporting mild pain was inversely associated with age, such that the older the patient, the less mild pain he or she reported. However, no difference was found across age groups associated with severe painful stimuli [12]. An alternative explanation for this phenomenon may be that older patients presented with more severe cases of COVID-19 and were subsequently more likely to be intubated (p = 0.044). The considerably compromised respiratory status likely took precedence, and the patient may not have reported less intense pain complaints.

Table 2 shows that specific pain complaints did not differ across age groups except for younger people reporting statistically more chest pain and headache than older populations (p = 0.005 and 0.020). In young patients presenting to their primary care doctor or emergency department with chest pain, COVID-19 must remain in the differential diagnosis. Acute coronary events in young people are rare. In a study of 487 patients aged <30 years who presented to the emergency department with chest pain and without preexisting cardiac disease or risk factors, patients had a <1% chance of an acute coronary event [13]. The chest pain reported in our study was often described as “sharp” and worse with coughing or deep breathing, typical of pleuritic chest pain. As noted in Table 3, there was a significant difference in the number of Native Americans/Pacific Islanders with headaches as a pain complaint. This is not clinically significant and likely an incidental finding in the setting of a disproportionate number of White patients compared to other races, a weakness of the study.

Table 4 highlights the abdominal pathologies found in those with abdominal pain who received a form of diagnostic imaging. One of the patients was found to have a newly diagnosed left ovarian serum mucinous cystadenoma as the possible source of her abdominal pain symptom. The other significant abdominal pathology elucidated with diagnostic imaging was in a patient with a newly diagnosed deep vein thrombosis in his lower inferior vena cava down to his bilateral lower extremity veins. The final significant pathology found with diagnostic imaging was in a patient with a CT abdomen and pelvis with contrast concerning constipation.

The other findings from imaging were nonspecific and not likely to be a source of abdominal pain. One of the possible explanations for why COVID-19 causes abdominal pain is its interaction with ACE2 receptors that are highly expressed in the esophageal and intestinal epithelium. COVID-19 binds to ACE2 receptors in the gastrointestinal tract, eliciting gastrointestinal symptoms such as diarrhea, nausea, emesis, and abdominal pain [14]. Bolia et al. noted increased abdominal pain presentation in children infected with COVID-19 compared to adults. They noted that 12% of pediatric patients that tested positive for COVID-19 had gastrointestinal symptoms that included clinical signs of pseudoappendicitis [15]. Although Mao et al. showed a comparable prevalence of gastrointestinal symptoms between pediatric and adult patients, they also found that COVID-19-infected patients with abdominal symptoms were more likely to have a critical disease and a poor disease course [16]. Patients who presented with gastrointestinal symptoms were also more likely to develop acute respiratory distress syndrome [16].

As the COVID-19 pandemic continues, further studies are needed to investigate pain in patients with COVID-19. Several studies have reported on post-COVID-19 persistent symptoms, encompassing fatigue, breathlessness, psychological disturbance, and overall reduced quality of life with an emphasis on pain [17,18]. In the same study, 28.1% of patients discharged from the intensive care unit reported worsened pain since disease onset at the seven-week discharge follow-up. Several studies have reported chronic pain as an atypical symptom some patients suffer after COVID-19 infection. Although the exact mechanism for developing post-COVID-19 pain syndrome is unknown, several risk factors include acute pain, extended proning, protracted ventilation, patient age, and overall physical condition [19,20]. If acute pain is left inadequately treated, pain can become chronic, leading to significant morbidity.

Limitations

This is a retrospective study with limitations typical of a retrospective study design. One of the primary limitations of the study is the sample size. The sample size was limited because of decreased hospital volume during the onset of the COVID-19 pandemic. Hence, a total of 104 patients seen at the emergency department from February 1, 2020, to April 27, 2020, had a positive COVID-19 PCR test. The study was conducted at a single emergency department. Thus, further investigation is needed to validate our result. Because different triage nurses are involved in the triage process, individual bias may occur while triaging patients. Moreover, patients might be biased in reporting common symptoms reported by news media as the presenting symptoms of COVID-19. Although a statistically significant number of patients with abdominal pain were intubated compared to patients without abdominal pain, other factors, such as advanced age and comorbid medical conditions, could have cofounded the study.

## Conclusions

In this study, we investigated different pain complaints in relation to demographics and outcomes, finding that abdominal pain in older patients is a risk factor for intubation. This finding is of clinical importance because the COVID-19 virus continues to be viewed as primarily a respiratory illness. The next step would be a prospective study investigating patients’ outcomes based on their presenting symptoms in the emergency department and other healthcare settings, such as primary care offices or specialty clinics.
